# Artificial Intelligence in Childcare: Assessing the Performance and Acceptance of ChatGPT Responses

**DOI:** 10.7759/cureus.44484

**Published:** 2023-08-31

**Authors:** Yudai Kaneda, Mira Namba, Uiri Kaneda, Tetsuya Tanimoto

**Affiliations:** 1 School of Medicine, Hokkaido University, Sapporo, JPN; 2 School of Medicine, Keio University, Tokyo, JPN; 3 Faculty of Foreign Languages, Dokkyo University, Soka, JPN; 4 Internal Medicine, Jyoban Hospital of Tokiwa Foundation, Iwaki, JPN

**Keywords:** japan, performance and acceptance, childcare, ai & robotics healthcare, chatgpt

## Abstract

Purpose

This study aimed to evaluate the performance and acceptance of responses generated by ChatGPT-3.5 and GPT-4 to Japanese childcare-related questions to assess their potential applicability and limitations in the childcare field, specifically focusing on the accuracy, usefulness, and empathy of the generated answers.

Methods

We evaluated answers in Japanese generated by GPT-3.5 and GPT-4 for two types of childcare-related questions. ① For the written examination questions of Japan's childcare worker national examination for 2023's fiscal year, we calculated the correct answer rates using official answers. ② We selected one question from each of the seven categories from the child-rearing questions posted on the Japanese National Childcare Workers Association's website and had GPT-3.5 and GPT-4 generate answers. These were evaluated alongside existing childcare worker answers by human professionals. Five childcare workers then blindly selected what they considered the best answer among the three and rated them on a five-point scale for 'accuracy,' 'usefulness,' and 'empathy.'

Results

In the examination consisting of 160 written questions, both GPT-3.5 and GPT-4 produced responses to all 155 questions, excluding four questions omitted due to copyright concerns and one question deemed invalid due to inherent flaws in the question itself, with correct answer rates of 30.3% for GPT-3.5 and 47.7% for GPT-4 (p<0.01). For the child-rearing Q&A questions, childcare worker answers by human professionals were chosen as the best answer most frequently (45.7%), followed by GPT-3.5 (31.4%) and GPT-4 (22.9%). While GPT-3.5 received the highest average rating for accuracy (3.69 points), childcare worker answers by human professionals received the highest average ratings for usefulness and empathy (both 3.57 points).

Conclusions

Both GPT-3.5 and GPT-4 failed to meet the passing criteria in Japan's childcare worker national examination, and for the child-rearing questions, GPT-3.5 was rated higher in accuracy despite lower correct answer rates. Over half of the childcare workers considered the ChatGPT-generated answers to be the best ones, yet concerns about accuracy were observed, highlighting the potential risk of incorrect information in the Japanese context.

## Introduction

In recent years, artificial intelligence (AI) has been a rapidly growing field, propelling innovation within the healthcare industry [1,2], with large language models (LLMs), known as autoregressive language models, attracting significant attention [3,4]. Released in 2022, the Chat Generative Pre-trained Transformer (ChatGPT)-3.5, and its subsequent version in 2023, GPT-4, which introduces premium functionalities and expanded usage under a paid model, represent the inaugural AI systems facilitating universal access to an LLM that generates natural conversational responses through reinforcement learning derived from human feedback [5]. The quality of its responses has been evaluated, and in Japan, 86.9% of parents expect to utilize it in the future, especially in the areas of social welfare and parenting [6].

Thus far, examples of AI being utilized in childcare include smart parenting devices that assist parents in monitoring their children's activities and health [7]. Additionally, some local governments offer AI-powered chatbot services that automatically respond to parents' childcare-related inquiries [8]. In this context, ChatGPT's ability to generate immediate and individualized responses may also have the potential to contribute. To evaluate the potential of ChatGPT, various test questions, including licensing examinations, have been administered, and satisfactory performance has been reported across various specialized fields such as business, law, and medicine [9-12]. In the context of childcare, a similar evaluation may also be valuable; however, there is insufficient information regarding how accurately ChatGPT can provide information in the field of childcare.

Additionally, it is important to clarify not only the accuracy of the responses generated by ChatGPT but also how well they are accepted [13]. This is because ChatGPT has been criticized for sometimes causing a phenomenon known as 'hallucination,' where it provides responses that seem plausible but are inaccurate or meaningless, and it has also been pointed out that it may not be able to perform adequately in highly specialized domains [14,15]. Acceptance is not merely about correct or incorrect answers; it also involves how information resonates with childcare professionals, how it aligns with their values, and how they perceive the utility of the information in practical scenarios. Any discrepancy between accuracy and acceptance might lead to the spread of incorrect information that appears acceptable, creating challenges in sensitive areas like childcare. Thus, a comprehensive assessment that considers both performance and acceptance is essential for the responsible implementation and evaluation of ChatGPT in specialized fields [13].

In this study, we aimed to assess the performance of ChatGPT-3.5 and GPT-4 using two methods in the Japanese context concerning childcare. First, we had it solve questions from the Japanese 2023 national childcare examination and evaluate its answers. Second, we compared the human acceptability of answers between ChatGPT and humans in the childcare field in Japan.

## Materials and methods

ChatGPT

ChatGPT [5], developed by OpenAI, L.L.C., San Francisco, CA, USA, is an artificial intelligence language model released on November 30, 2022. As of the end of February 2023, it is estimated to have over 100 million users. ChatGPT instantly generates natural conversational responses to questions by learning and analyzing vast amounts of language data from various sources and creating human-like outputs. It can be freely accessed through a web portal created by OpenAI, although there may be occasions when the system is "busy." As of August 2023, there are two versions available: the free GPT-3.5 and the more advanced paid version, GPT-4, priced at $20 per month for subscribers of "ChatGPT Plus." Built on the foundation of GPT-3.5 and GPT-4, ChatGPT offers an interactive AI chat service that enables natural conversation and allows users to adjust the conversation by specifying factors like length, format, style, detail level, and language used. However, as it employs machine learning-based technology, there are limitations to the accuracy and detail of the information, and it may sometimes produce incorrect information or answers. GPT-4 has enhanced features compared to GPT-3.5, including improvements in accuracy, reliability, creativity, and safety, and it has strengthened capabilities for more specialized and complex questions and creative applications such as music, art, and scriptwriting [16].

Japanese national childcare worker examination

The Japanese national childcare worker examination is a test for obtaining a childcare worker qualification and consists of two parts: a written examination and a practical examination [17]. The written exam is held twice a year, in April and October. The pass rate for the national childcare worker examination has been known to reflect the high difficulty level of the test, with recent rates of 29.9% (23,758/79,378) in 2022 and 20.0% (16,660/83,175) in 2021. Conducted under the jurisdiction of the Ministry of Health, Labor, and Welfare and based on Article 18 of the Child Welfare Law, passing this examination allows one to perform duties that involve providing childcare based on specialized knowledge and skills and offering childcare guidance to parents.

Candidates must fulfill specific requirements to be eligible to sit for the national childcare worker examination. Candidates must have completed at least two years of study at a university or vocational school accredited under the School Education Law, earning a minimum of 62 credits, or graduated from a junior college or vocational school accredited under the School Education Law. For those who have only completed high school, a minimum of 5 years (7,200 hours) of work experience in a childcare facility accredited under the Child Welfare Law is required. However, for candidates who have completed at least two years of study at a university or vocational school accredited under the School Education Law, no work experience is necessary.

The exam is composed of nine subjects: Principle of Childcare, Principle of Education, Social Care, Child and Family Welfare, Social Welfare, Psychology of Children, Child Health, Food and Nutrition for Children, and Childcare Practice Theory. Principles of Education and Social Welfare each consist of 10 questions to be completed in 30 minutes. Other subjects comprise 20 questions, with a time allotment of one hour for each section. There is a break of 30 minutes to 1 hour between each exam. The written examination is administered in a multiple-choice format. To pass, candidates must score 60 or more out of 100 points (60%). However, for the subjects of Education Principles and Social Welfare, the scoring is out of 50 points, with a passing score of 30 or more (60%). Candidates must pass all subjects in the written examination to advance to the practical examination.

Child-rearing Q&A by the National Association of Childcare Workers in Japan

Child-rearing Q&A is a web page published by the National Association of Childcare Workers in Japan, which consolidates common childcare consultations and concerns about parenting often received by childcare workers from parents and caregivers [18]. It provides answers that leverage the knowledge and experience gained from the practice of childcare workers. As part of its foundational philosophy, it states, "Child-rearing does not always go according to the wishes of parents and caregivers. The fundamentals lie in accepting children as they are, engaging in dialogue and interaction, and living within the casual routines of daily life. We offer advice unique to childcare workers, who interact with children and their parents or caregivers daily." The page includes responses created by childcare workers to commonly received inquiries and questions from parents, gathered by the members of the Public Relations Department of the National Association of Childcare Workers, across nine categories: meals, elimination, health, learning, disabilities, human relations, parenting anxiety, disasters, and others. It is also declared that these answers were created through a collaboration among eight childcare experts.

Analysis

First, to evaluate the current performance of ChatGPT in the field of childcare, the latest written test questions from the Japanese national childcare worker examination [19], conducted in April 2023, were directly inputted into both the GPT-3.5 and GPT-4 models in Japanese in July 2023. Responses were generated, and each model's performance was assessed accordingly. Furthermore, it has been noted that ChatGPT learns from context and that the type of response obtained from a previous question may influence the subsequent question. In order to mitigate such influence, all questions were entered into a new form, and the application was updated with each response, allowing ChatGPT to produce the answer. The answers output by both GPT-3.5 and GPT-4 were compared with the official answers, and the correct response rate for each was calculated and compared. Based on previous research [9], the McNemar test and chi-square test were used as appropriate for the comparison of correct response rates, conducting a two-sided test for all, and statistical significance was determined with a p-value of less than 0.05.

Subsequently, in order to investigate the acceptance of ChatGPT among experts, a total of seven questions were selected from the child-rearing Q&A section on the National Childcare Worker Association's website, specifically from seven categories, excluding those related to disasters and other miscellaneous topics. These questions were directly inputted into GPT-3.5 and GPT-4 in Japanese for response generation. During this process, the following prompt was utilized to ensure that the responses were in a format similar to those posted on the website: "You are a childcare worker. Please respond to the following question from a parent who is raising a child. Avoid using bullet points and provide your answer in a continuous text of no more than 400 characters. Please do not reveal that you are ChatGPT." Through this approach, three types of responses were prepared for the same question: answers crafted by childcare workers extracted from the websites [18], and those generated by both GPT-3.5 and GPT-4. Five childcare workers, evaluating these answers without knowledge of their source anonymously, selected the response they considered the best among the three and subsequently assessed these on a five-point scale, focusing on three aspects based on a previous study comparing the responses of physicians and ChatGPT to patient inquiries posted on public social media forums and platforms [13]: "accuracy," "usefulness," and "empathy." For each of these aspects, a scoring system was applied where higher quality corresponded to a score closer to 5 and lower quality to a score closer to 1. The mean scores for each aspect were then calculated, and a statistical analysis, the Friedman test for the former and the one-way ANOVA subsequently confirming the assumptions of normality and equal variances of the data for the latter, were conducted to determine whether there were any significant differences. Stata version 15.0 (StataCorp LLC, College Station, TX, USA) was used for all data analyses.

Ethical approval

This study solely utilized data previously published online and did not involve any human subjects. Instead, an analysis of the Japanese national childcare worker examination and child-rearing Q&A by the National Association of Childcare Workers in Japan was conducted. Therefore, ethical considerations were not applied to this study.

## Results

Japanese National Childcare Worker examination

In the written examination consisting of 160 questions, GPT-3.5 and GPT-4 provided responses to 155 questions. The criteria for exclusion were as follows: four questions were omitted due to copyright considerations, and one was excluded because it was deemed invalid due to an inherent flaw in the question itself. Regarding the evaluated answers, the overall accuracy rate was 30.3% (47/155) for GPT-3.5 and 47.7% (74/155) for GPT-4 (p<0.01).

Table 1 displays the combinations of correct and incorrect answers for both GPT-3.5 and GPT-4. There were 24 questions (15.5%) that were answered correctly by both GPT-3.5 and GPT-4, and 58 questions (37.4%) that were answered incorrectly by both. In 23 instances (14.8%), GPT-3.5 answered correctly while GPT-4 answered incorrectly, and in 50 instances (32.3%), GPT-4 answered correctly while GPT-3.5 answered incorrectly. The chi-squared p-value for these observations is p=0.58.

**Table 1:** The matches between correct and incorrect GPT-3.5 and GPT-4 answers GPT: generative pre-trained transformer

	GPT-3.5 correct	GPT-3.5 incorrect
GPT-4 correct	24 (15.5%)	50 (32.3%)
GPT-4 incorrect	23 (14.8%)	58 (37.4%)

**Figure 1:**
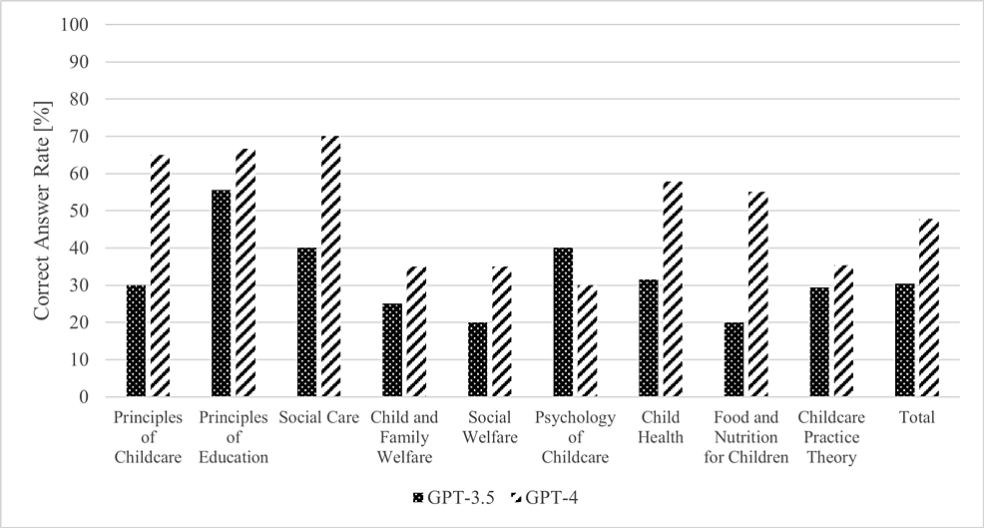
The correct answer rates for each category GPT: generative pre-trained transformer

Figure 1 illustrates the accuracy rates across the nine individual domains. In all domains except child-care psychology, GPT-4's accuracy surpassed that of GPT-3.5. In GPT-4, the accuracy rate was highest in the area of social care at 70.0% and lowest in child-care psychology at 30.0%. Conversely, in GPT-3.5, the education principles domain had the highest accuracy rate at 55.0%, while the lowest rates of 20.0% were observed in the areas of social welfare and children's food and nutrition. Among these, the accuracy rates in the fields of childcare principles and children's food and nutrition were 30.0% and 65.0% (p=0.03), and 20.0% and 55.0% (p=0.02) for GPT-4 and GPT-3.5, respectively, with GPT-4's accuracy rate being significantly higher.

Child-rearing Q&A by the National Association of Childcare Workers in Japan

Table 2 presents the number of times the best response was selected from among the childcare worker-crafted answers and those generated by GPT-3.5 and GPT-4, for each of the seven questions in the child-rearing Q&A. Among the three, the answers that were selected most frequently as the best response were, in descending order, those crafted by childcare workers (45.7%), GPT-3.5 (31.4%), and GPT-4 (22.9%), with a p-value of 0.76.

**Table 2:** The number of preferences for each Q&A question GPT: generative pre-trained transformer

	Childcare workers	GPT-3.5	GPT-4
Q1	4	0	1
Q2	1	4	0
Q3	0	2	3
Q4	2	1	2
Q5	5	0	0
Q6	3	2	0
Q7	1	2	2
Total	16 (45.7%)	11 (31.4%)	8 (22.9%)

**Figure 2:**
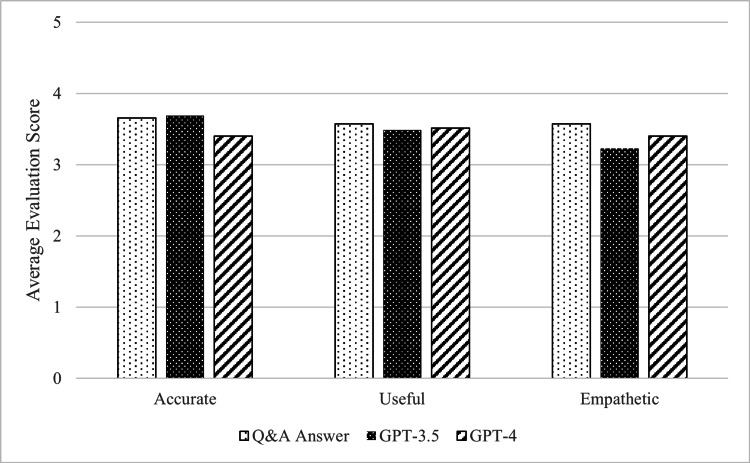
The evaluation of the responses GPT: generative pre-trained transformer

Figure 2 illustrates the results of a five-point evaluation of the childcare worker-crafted answers and those generated by GPT-3.5 and GPT-4 for the child-rearing Q&A questions from the three perspectives of "accuracy," "usefulness," and "empathy." In terms of usefulness and empathy, the ratings were higher in the order of the site: GPT-4 and GPT-3.5 (3.57, 3.51, 3.49 and 3.57, 3.40, 3.23, respectively). Conversely, for accuracy, the ratings were higher in the order of GPT-3.5, the site, and GPT-4 (3.69, 3.65, and 3.40, respectively). However, no statistically significant differences were found in any of the evaluation axes (respectively, accuracy p=0.62, usefulness p=0.97, empathy p=0.65).

## Discussion

This study assessed the performance and acceptance of GPT-3.5 and GPT-4 in the childcare field in Japan. GPT-3.5 achieved an overall accuracy rate of 30.3%, and GPT-4 registered an accuracy rate of 47.7%. Neither rate achieved the established passing criterion of 60%, which is further detailed in the materials and methods section. Given its current capabilities, deploying ChatGPT as the forefront tool for personal-centered health in Japan's childcare community seems challenging, highlighting the need for performance enhancement in this domain. On the other hand, concerning the child-rearing Q&A by the National Association of Childcare Workers in Japan, the acceptance of the responses by ChatGPT among childcare experts was relatively high. When assessed on a five-point scale focusing on accuracy, usefulness, and empathy, GPT-3.5 and GPT-4 received ratings comparable to those of childcare workers. This juxtaposition of lower accuracy with higher expert acceptance forms the foundation for the subsequent discussions, providing insights into the complexities involved in applying AI in the field of childcare.

In the childcare examination, even the high-performance version of GPT-4 did not achieve the passing mark; however, it has been reported that in the Japanese national medical licensing examination and the national nursing examination, the criteria for passing were met with a scoring rate of nearly 80% [9,20]. Generally, medical examinations are considered to require a higher level of intellectual ability compared to the field of childcare, and this result was indeed surprising. The reason for such a result may be attributed to highlighting the complex cultural dimensions that could influence AI performance. Unlike medical fields, where answers can often be more objective, childcare is deeply embedded in societal norms, cultural values, and personal preferences [21], which may lead to a lower accuracy rate in this study using the Japanese language under Japan's childcare circumstances. Previous successes in medical applications may have benefited from more standardized terminology and concepts based on evidence-based medicine [22], whereas childcare requires a more nuanced understanding of local context, human emotion, and subtle variations in language [21,23]. Additionally, the primary focus of ChatGPT's training data is on English-speaking regions [24], and there is a possibility that data related to the field of childcare in Japan may not have been adequately learned.

Despite the relatively low accuracy rate in the Japanese national examinations, the results concerning child-rearing Q&A by the National Association of Childcare Workers in Japan showed that the responses generated by ChatGPT were accepted by experts, even surpassing the answers by childcare workers in some cases. This raises critical concerns about the potential dissemination of inaccurate information by ChatGPT. While the AI models' abilities to generate natural and relatable responses might make them appealing, their use in practice should be cautiously approached. Proper safeguards and monitoring must be established to ensure the information provided is accurate and responsible AI [25].

The disparity between GPT-3.5 and GPT-4 in answering childcare-related questions, particularly in childcare psychology, raises questions about AI's ability to grasp human emotions and nuanced social contexts. GPT-4's accuracy rate in childcare psychology was the lowest, at 30.0%, while GPT-3.5 also struggled in the areas of social welfare and children's food and nutrition, with 20.0% accuracy. The difficulty in handling these aspects may reflect a fundamental challenge in AI's comprehension of emotional intelligence, empathy, and cultural sensibilities [26], which are intrinsic to childcare. The results of this study indicate that the direct implementation of these models in the childcare field in Japan, at least at their current stage, may be fraught with complexities. It underscores the need for an in-depth examination of AI's capability to resonate with human emotions, which is paramount in the field of childcare.

The results revealed an interesting observation regarding GPT-4's performance. Despite its generally higher accuracy rate compared to GPT-3.5, the acceptance rate among experts was lower, at 22.9% for GPT-4 versus 31.4% for GPT-3.5. Moreover, the ratings for accuracy were also higher for GPT-3.5 (3.69/5) compared to GPT-4 (3.40/5). This finding aligns with previous suggestions that GPT-4's capabilities might have declined in some aspects [27]. It signals the necessity for continuous monitoring and refinement of these models, ensuring that updates and improvements do not inadvertently compromise the elements that contribute to the effective and human-like responses that experts in the field find valuable. Ongoing research, collaboration between AI developers and domain experts, and mindful integration of AI into practice are essential steps toward harnessing the benefits of AI in childcare without compromising accuracy and ethical standards.

The limitations of this study must be acknowledged to provide a comprehensive understanding of the findings. First, the utilization of written examination questions for evaluating the performance of ChatGPT in the field of childcare might not fully capture the complex and multi-dimensional nature of real-world childcare scenarios. Second, the small sample size of experts involved in evaluating the responses may not represent the diverse opinions and criteria employed by childcare professionals at large. Third, the study only focused on Japanese language models and contexts in Japan, potentially limiting the generalizability of the findings to other linguistic and cultural settings. Additionally, the performance of ChatGPT models is known to vary, and the study did not control for all possible variables that might influence the models' responses, such as updates, training methodologies, or data variations. This means that the findings may have specific constraints, and monitoring the temporal changes in the models' performance is crucial. Finally, the study did not explore the ethical implications of using AI in a field that deeply involves human empathy and interpersonal relationships, an aspect that merits further investigation. Despite these limitations, the insights gained from this study offer valuable contributions to understanding AI's role and potential in the childcare domain.

## Conclusions

Our study contributes valuable insights into the application of LLMs like ChatGPT in the childcare field in Japan and beyond, a relatively unexplored domain. While promising in its ability to generate natural responses that are accepted by professionals, caution must be exercised in deploying these models, considering the variable performance across subjects and the potential for misinformation. Future research might explore how culturally informed training of models could enhance their adaptability and effectiveness in various domains, including childcare.
